# Comparison of 3D Bone Position Estimation Using QR Code and Metal Bead Markers

**DOI:** 10.3390/diagnostics13061141

**Published:** 2023-03-16

**Authors:** Akira Ikumi, Yuichi Yoshii, Yuta Iwahashi, Satoshi Sashida, Pragyan Shrestha, Chun Xie, Itaru Kitahara, Tomoo Ishii

**Affiliations:** 1 Department of Orthopaedic Surgery, Tsukuba University Hospital, Tsukuba 305-8576, Japan; 2 Department of Orthopaedic Surgery, Tokyo Medical University Ibaraki Medical Center, Ami 300-0395, Japan; 3 LEXI Co., Ltd., Sugamo, Tokyo 170-0002, Japan; 4 Center for Computational Sciences, Tsukuba University, Tsukuba 305-8577, Japan

**Keywords:** 3D, tracking, computed tomography, fluoroscopy, QR code, preoperative plan, distal radius fracture

## Abstract

To improve the accuracy of a 3D bone position estimation system that displays 3D images in response to changes in the position of fluoroscopic images, modified markers using quick response (QR) codes were developed. The aims of this study were to assess the accuracy of the estimated bone position on 3D images with reference to QR code markers on fluoroscopic images and to compare its accuracy with metal bead markers. Bone positions were estimated from reference points on a fluoroscopic image compared with those on a 3D image. The positional relationships of QR code and metal bead markers on the fluoroscopic image were compared with those on the 3D image in order to establish whether a 3D image may be drawn by tracking positional changes in radius models. Differences were investigated by comparing the distance between markers on the fluoroscopic image and that on the 3D image, which was projected on the monitor. The error ratio, which was defined as the difference in the measurement between the fluoroscopic and 3D images divided by the fluoroscopic measurement, was compared between QR code and metal bead markers. Error ratios for the QR code markers were 5.0 ± 2.0%, 6.4 ± 7.6%, and 1.0 ± 0.8% in the anterior–posterior view, ulnar side lateral view, and posterior–anterior view, respectively. Error ratios for the metal bead markers were 1.3 ± 1.7%, 13.8 ± 14.5%, and 4.7 ± 5.7% in the anterior–posterior view, ulnar side lateral view, and posterior–anterior view, respectively. The error ratio for the metal bead markers was smaller in the initial position (*p* < 0.01). However, the error ratios for the QR code markers were smaller in the lateral position and the posterior–anterior position (*p* < 0.05). In QR code marker tracking, tracking was successful even with discontinuous images. The accuracy of a 3D bone position estimation was increased by using the QR code marker system. QR code marker tracking facilitates real-time comparisons of dynamic changes in preoperative 3D and intraoperative fluoroscopic images.

## 1. Introduction

Three-dimensional pose estimation is one of the most active topics in computer vision research. Effective algorithms that use 2D–3D point correspondences between pairs of images have been developed in several ways [1,2]. However, these techniques cannot be directly applied to transmission images (i.e., fluoroscopic images) because of complications caused by inconvenient calibration objects or the failure of feature matching algorithms. A general goal of 2D–3D registration is to establish geometric transformation between the coordinate system of a 3D object and that of a device, such as a camera that captures a 2D image. In clinical use, it is important to align the 3D model of an anatomical structure with a corresponding 2D radiographic image, which is typically obtained from a regular X-ray, computed tomography (CT), or interventional fluoroscopy [3]. For example, orthopedic surgeons use fluoroscopy to evaluate the reduction shape of the fractured bone during osteosynthesis. The 2D fluoroscopic image is replaced in the surgeon’s head with a 3D model to estimate the reduction shape’s accuracy. In such a situation, the estimation of bone directions is necessary. Sometime, this causes a difference in surgical outcomes between experienced and inexperienced operators.

Registration methods for 2D–3D images have been developed with several different protocols [4,5,6,7,8,9]. Previous studies used edge-enhanced images of CT data [5], single- or bi-plane X-ray imaging with model-based shape matching [4], or projection images with tomosynthesis [9]. These methods were found to be beneficial under conditions in which stable bone imaging was possible. On the other hand, the establishment of techniques that align 3D images with fluoroscopic images of structures that significantly move or deform during surgery is needed, particularly in cases of fracture reduction or bone osteotomy. Physicians need to confirm the position of the reduction or internal fixation installation from various directions during these operations.

We previously developed a 3D bone position estimation system that displays 3D images created before surgery in response to changes in the positions of metal bead markers on 2D dynamic fluoroscopic images during surgery [10]. The 3D bone image showed higher accuracy in the anterior–posterior and posterior–anterior views than in the lateral view. Although this system tracked the rotational motion of the target tissue with an error of less than 3 mm under fluoroscopy, difficulties were associated with identifying the positional relationship of the markers in the lateral view and with tracking discontinuous images. To improve the accuracy of identifying the positional relationships of markers in the lateral view, we developed a new technique using a quick response (QR) code as a marker. A QR code is a type of matrix barcode (or two-dimensional barcode) invented in 1994 by the Japanese automotive company Denso Wave [11]. A barcode is a machine-readable optical label that contains information on the item to which it is attached. In practice, QR codes often contain data for a locator, identifier, or tracker that points to a website or application. QR codes use four standardized encoding modes (numeric, alphanumeric, byte/binary, and kanji) to efficiently store data; extensions may also be used. QR codes became popular due to their fast readability and greater storage capacity. Applications include product tracking, item identification, time tracking, document management, and general marketing.

In the present study, we hypothesized that QR code markers may improve the tracking of fluoroscopic images over that with regular metal bead markers. Therefore, we assessed the accuracy of the estimated bone position in 3D images with reference to QR code markers on fluoroscopic images and compared its accuracy with metal bead markers.

## 2. Materials and Methods

**Figure 1:**
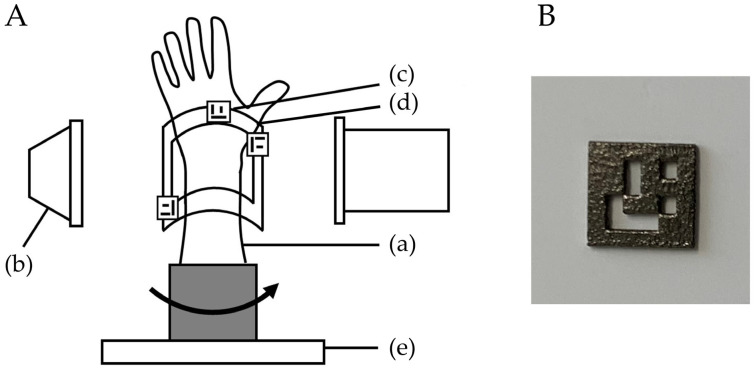
(**A**) Experimental setting of bone model using QR code markers. (**a**) Bone model. Bone models were covered with an X-ray-transparent, elastic material that imitated skin. (**b**) Position of the image intensifier for fluoroscopy. (**c**) QR code marker. (**d**) Splint to position the markers. The splint was attached to the bone model. (**e**) Turntable to rotate the bone model. (**B**) QR code marker made of titanium.

This study protocol was approved by our Institutional Review Board (T2019-0178). This was an experimental study on bone models. A normal wrist bone model was evaluated using the bone position estimation system. A custom-made bone model was prepared based on the CT data of a previous case with a normal wrist. By using CT data, the bone model was made from epoxy resin, which can be visualized with fluoroscopy (TANAC Co., Ltd., Gifu, Japan). The bone model was covered with an X-ray-transparent, elastic material (urethane resin) that imitated skin. The system estimated the 3D position of the radius by comparing the reference markers of QR codes or metal beads on a fluoroscopic image with markers on a 3D image created from CT images. The experimental setting using QR code markers is shown in Figure 1.

The bone model was placed on a turntable to imitate the rotational movement of the forearm. To evaluate the accuracy of the 3D position of the bone in the fluoroscopic image, a splint with three QR code or four metal bead markers was placed on the radius bone model as a reference point, and CT scans were performed. CT images were taken with a tube setting of 120 kV and 100 mAS, a section thickness of 0.8 mm, and a pixel size of 0.3 × 0.3 mm (Sensation Cardiac, Siemens, Berlin, Germany). Three-dimensional bone images of the forearm models were created from the DICOM datasets of CT scans. Image analysis software (ZedView, LEXI Co., Ltd., Tokyo, Japan) was used to create a 3D bone image [12]. After importing image data into the software, 3D images were created by extracting the bone lesion and reference points. A distal radius 3D model was created by extracting the area of the radius. The bone model was then visualized with fluoroscopy (Cios Select, Siemens, Berlin, Germany). The C-arm fluoroscopy system was placed perpendicular to the bone model, and the model was rotated to depict the bone image. The bone model was placed on a turntable in the center of the X-ray output unit. The tracking of positional changes in the bone model was verified by half rotation of the turntable (mimicking a surgical situation). The placement of the bone model was reproduced by pasting tape so that the center of the turntable and the center of the bone model were in the same position. Bone positions were estimated from the reference markers on the fluoroscopic image by comparisons with those on the 3D image.

### 2.1. Three-Dimensional Position Estimation System

**Figure 2:**
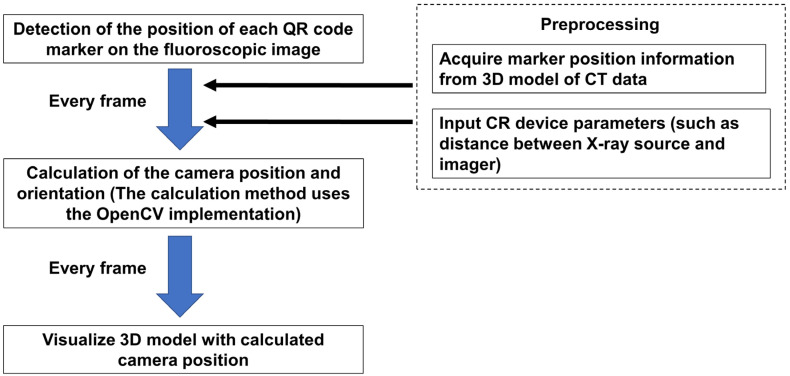
Algorithm of the QR code marker tracking.

We used the 3D position estimation and tracking program to detect reference points on the screen and track the motion of a fluoroscopic image as described by Yoshii et al. [10]. This system is a program that outputs fluoroscopic images to a computer and can be operated on the computer. In the present study, it was set to recognize the metal bead and QR code markers as the reference points. We used a splint to reproduce each marker position. The program was set up to track pre-specified reference markers. The metal bead marker tracking algorithm has been described previously [10]. In brief, the program is configured to automatically extract candidate marker points from the image and calibrate them by relating them to points on the 3D image. The system monitored fluoroscopy images and extracted candidate markers from each frame. A linear interpolation of the estimated positions from the last five frames was performed to determine the set of metal marker positions. Based on the results of linear interpolation, positions of the reference point for the next frame were estimated. The system compared the relative positions of the markers on the perspective image of the current frame with the relative positions of the reference markers on the 3D model. Then, the camera pose (orientation, position) closest to the positional relationship of the fiducial markers on the fluoroscopic image was calculated. Finally, a 3D image corresponding to the viewpoint of the 3D model was displayed. The QR code marker tracking algorithm is shown in Figure 2. As part of the preparation, the marker position information was acquired from the 3D model of the CT data. Then, the fluoroscopy conditions (such as distance between X-ray source and imager) were entered into the program. In the beginning, the program detected the position of each QR code marker on the fluoroscopic image. According to the preparation information, the camera position and orientation were calculated. The calculation method uses the open-source computer vision library (OpenCV) for implementation [13,14]. These processes were run on each frame to visualize the 3D model with the calculated camera positions. Table 1 shows the difference in the algorithm of the tracking.

### 2.2. Evaluations

**Figure 3:**
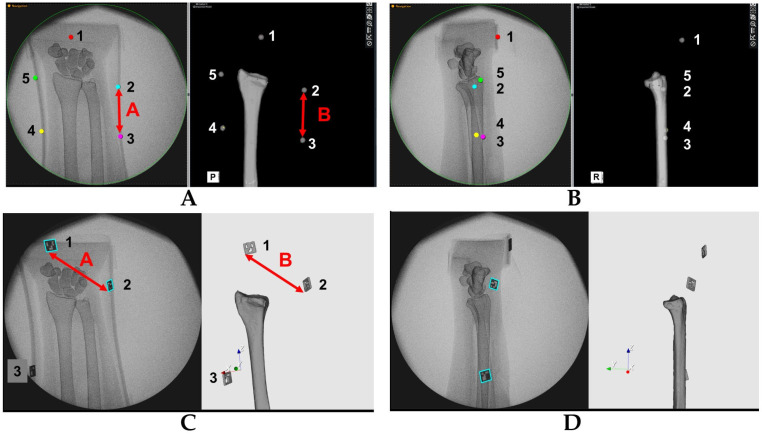
Evaluations of the tracking accuracy. Example images of the tracking. (**A**) Anterior–posterior view and (**B**) ulnar side lateral view with metal sphere markers, and (**C**) anterior–posterior and (**D**) ulnar side lateral view with QR code markers. A and B in the figures show the distance between markers for the fluoroscopic and 3D images. The numbers in the figures indicate the marker number. Accuracies were evaluated by comparing the distance between markers on the fluoroscopic and 3D images, which were projected on the monitor.

To evaluate the accuracy of the estimated 3D position of bone models, the positions of the metal bead and QR code markers on the fluoroscopic image and on the created 3D bone image were compared (Figure 3). We verified whether the 3D bone image may be drawn by tracking positional changes in the forearm model. Accuracies were investigated by comparing the distance between markers on the fluoroscopic image (A) and on the 3D image (B), which were projected on the monitor. The center of the metal bead marker and a specific edge of the QR code marker were defined as measurement points. The distances between markers were measured using ImageJ software 1.53 (NIH, Bethesda, MD, USA). After importing the images into the software, measurements were performed under the following three conditions: anterior–posterior view, ulnar side lateral view, and posterior–anterior view. Differences in the distance of markers on the fluoroscopic image and on the 3D image (= A–B) were evaluated at each position. Distances were measured between each marker (1–2, 2–3, 3–4, 4–5, and 5–1 for metal sphere markers, and 1–2, 2–3, and 3–1 for QR code markers). For the metal sphere marker measurements, the center of each marker was identified on the monitor and the distance between the markers was measured. For the QR code marker measurements, the lower left corners of the markers were identified on the monitor and the distance between the markers was measured. The measurements were performed using five different fluoroscopic images for each marker. The average differences between markers for each position were calculated. The error ratio of the measurements between fluoroscopic and 3D images was defined as (A–B) × 100/A (%). Welch’s *t* test was used to evaluate the difference for the error between metal sphere and QR code markers. In addition, the intraclass correlation coefficients (ICCs) of the measurements between the fluoroscopic image and 3D bone image were assessed for all measurements. All results were expressed as mean ± standard deviation. Measurements were considered to be significant when the *p*-value was less than 0.05. All analyses were performed using SPSS Statistics (IBM, Tokyo, Japan) software.

## 3. Results

In QR code marker tracking, the differences between the measured values of the fluoroscopic image and the 3D bone image for the distance between each marker corresponding to the direction of the bone model were 5.0 ± 2.0 mm, 3.5 ± 3.2 mm, and 1.0 ± 1.0 mm in the anterior–posterior view, ulnar side lateral view, and posterior–anterior view, respectively. The error ratios were 5.0 ± 2.0%, 6.4 ± 7.6%, and 1.0 ± 0.8% in the anterior–posterior view, ulnar side lateral view, and posterior–anterior view, respectively.

In metal bead marker tracking, the differences between the measured values of the fluoroscopic image and the 3D bone image for the distance between each marker corresponding to the direction of the bone model were 0.9 ± 1.0 mm, 5.5 ± 4.3 mm, and 3.2 ± 4.2 mm in the anterior–posterior view, ulnar side lateral view, and posterior–anterior view, respectively. The error ratios were 1.3 ± 1.7%, 13.8 ± 14.5%, and 4.7 ± 5.7% in the anterior–posterior view, ulnar side lateral view, and posterior–anterior view, respectively.

**Figure 4:**
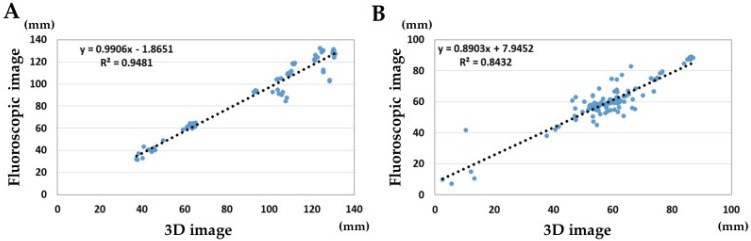
Correlations of marker distances between fluoroscopic image and 3D image. (**A**) QR code marker measurements. (**B**) Metal bead marker measurements. The blue dot indicate the measurements for the fluoroscopic and 3D images.

The error ratio for the metal bead markers was smaller in the anterior–posterior position compared to the error ratio for the QR code marker (*p* < 0.01). The error ratios for the QR code markers were smaller in the lateral position and the posterior–anterior position compared to the error ratios for the metal bead markers (*p* < 0.05). The ICCs of marker distances between the fluoroscopic image and the 3D image were 0.97 and 0.91 in the QR code marker tracking (Figure 4). There were better correlations among the QR code markers than among the metal bead markers.

In the metal bead marker tracking, tracking failed in the discontinuous images. In the QR code marker tracking, tracking was successful even when there were discontinuities in the images (total number of frames evaluated: 969, number of frames with successful marker detection: 872, success rate for the QR code marker detection: 90%).

## 4. Discussion

In this study, we found that there was better accuracy with the metal bead markers in the initial position for the fluoroscopic-image-based 3D bone position estimation system. However, there were better accuracies for tracking with the QR code markers after rotation. Three-dimensional bone position estimation systems using metal bead markers have been reported to have inferior localization accuracy in the lateral views compared to the anterior–posterior and posterior–anterior views [10]. The reason for the inferior accuracy in the lateral view is that the depth of the object cannot be detected from a two-dimensional perspective, and the proximity of the markers in the lateral view causes the distances between the markers on the 2D image to appear smaller than the actual distance. Additionally, the shape of the metal bead markers can also affect the detection of the distance between markers. Since the metal bead marker is a sphere, it is necessary to set the extraction point of the marker at the center of the marker. Therefore, it is possible that the front and back of the marker may not be recognized, resulting in a large measurement error. On the other hand, since the QR code marker can set a specific pattern of the marker as the detection point, it is thought that the front and back of the markers can be recognized and the position can be estimated even from the lateral-view image, thereby decreasing the measurement error.

In this study, QR code markers were able to track 3D images even in discontinuous images. The QR code markers we created this time were made of titanium, and the design of the code was simple so that it can be easily recognized even in the fluoroscopic images. Unlike the human body, which has low X-ray permeability, the QR code marker can be clearly visualized with ordinary fluoroscopy, and the recognition sensitivity of the 3D bone position estimation system was extremely high. Since the use of fluoroscopy during surgery poses the problem of radiation exposure to the operator and assistants, it is recommended to use it for short periods of time or in the pulse mode. The use of QR code markers enables accurate tracking and real-time 3D bone position estimation even with discontinuous fluoroscopic images taken in a short time, so we believe that problems in clinical application can be solved.

Attempts at 2D–3D registration of preoperative 3D images and fluoroscopic images for intraoperative navigation have been reported in various methods [8,9,15,16,17,18,19]. Many of these studies use either initial calibration, geometry approximated from source–detector distances recorded in the image data, or geometry measured by built-in measurement devices. Among orthopedic surgeries, 2D–3D registrations have been applied in spinal instrumentation surgery and joint replacement surgery in clinical practice [8,15]. In orthopedic trauma surgery, such as osteosynthesis in fracture surgery, the affected area is greatly deformed and moved during the surgery, so there is no established method for 2D–3D registration at the surgical site. Two-dimensional fluoroscopy images are still used as the gold standard for intraoperative evaluation [20,21,22,23]. Dynamic fluoroscopy images have been used for the multidirectional confirmation of bone conditions. The development of a system that can track the positional changes at a surgical site is required. The QR code marker we developed has a flat structure and can be sterilized. Therefore, it is thought that this can be applicable with few obstacles in clinical settings.

This study has some limitations. First, this study used simulated bone and placed markers on a self-made splint rather than on the skin surface. In actual surgery, it may be difficult to place a marker through a splint, and the condition of the soft tissue around the fractured area may change after trauma. In the future, it is necessary to verify the accuracy of this 3D bone position estimation system in a form that is more suitable for clinical application. Second, marker placement may be difficult depending on the condition of the surgical site. It is more desirable that alignment be performed without markers. Third, it was found that, when this system was used for large bones such as the pelvis, there was a discrepancy between the fluoroscopic image and the 3D image. This is due to the difference in magnification between sites that are near to or far from the C-arm and the difference in image distortion between the center and periphery of the irradiation field. For large bones, it is necessary to set more markers and develop a program that can track and estimate the positional relationship of some markers on the fluoroscopic images. These points need to be addressed in future research. Fourth, the accuracy of the initial position estimation using the QR code markers was inferior to that of the metal bead markers. This is because the actual measurements of the distance between markers were larger for the QR code markers than for the metal bead markers (average measurements of actual distance between QR code markers were 95.6 ± 31.1 mm and 95.6 ± 33.2 mm for the fluoroscopic image and the 3D image, respectively; average measurements of actual distance between metal bead markers were 60.9 ± 16.3 mm and 62.0 ± 16.9 mm for the fluoroscopic image and the 3D image, respectively). Finally, there were larger differences in the tracking accuracy compared to the previous study [10]. This is due to the differences in the number of frames of the analyzed images. In the previous study, there were about 600–900 image frames for each image. However, in this study, the image frames were only about 200. To reproduce a real surgical situation, we moved the bone model faster than the previous study did. This increased the inaccuracy of the tracking compared to the previous study.

## 5. Conclusions

In conclusion, the use of QR code markers reduces measurement errors during motion of 3D bone position estimation system. Furthermore, 3D image tracking is possible using QR code markers even in discontinuous images. The system may be useful for the real-time comparison of dynamic changes between preoperative 3D images and intraoperative fluoroscopic images.

## Figures and Tables

**Table 1:** Difference in the algorithm of the tracking for metal bead markers and QR code markers.

Metal Beads Marker	QR Code Marker
Unable to distinguish between metal beads markers ➡ Correspondence must be inferred from past marker positions	The QR code markers can be distinguished from patterns ➡ Can be calculated from a single fluoroscopic image
Use previous frame information to calibrate metal markers ➡ Only continuous images can be tracked	Calculate marker positions at each frame ➡ Tracking is possible even with discontinuous image sequences
Since the marker is a sphere, it can be detected from any direction as long as it does not cover the bone or implant ➡ Care must be taken in the installation position so that the marker does not cover the bone or implant	The pattern of the AR marker cannot be determined depending on the shooting direction ➡ Care must be taken with the placement position of the markers so that the markers do not face in the same direction

## Data Availability

The datasets analyzed during the present study are available from the corresponding author upon reasonable request.
